# Establishment and characterization of murine models of asthma and subcutaneous immunotherapy for *Humulus* pollen allergy

**DOI:** 10.1002/iid3.405

**Published:** 2021-01-12

**Authors:** Guang P. Xi, Qian Zhang, Jia Yin

**Affiliations:** ^1^ Department of Allergy, Peking Union Medical College Hospital, Chinese Academy of Medical Sciences Peking Union Medical College Beijing China; ^2^ Beijing Key Laboratory of Precision Medicine for Diagnosis and Treatment on Allergic Diseases Beijing China; ^3^ National Clinical Research Center for Dermatologic and immunologic Diseases Beijing China

**Keywords:** *Humulus* pollen, IP route, murine model of asthma, SC route, subcutaneous immunotherapy

## Abstract

**Introduction:**

*Humulus* pollen is an important cause of allergic asthma in East Asia. There have been some murine models for *Humulus* pollen allergy established by intraperitoneal (IP) sensitization and nasal drip stimulation, but they were not comprehensive enough. Here, we used atomized inhalation for challenge and compared the subcutaneous (SC) and IP sensitization routes to determine the optimal method to establish a model of asthma induced by *Humulus* pollen. Subsequently, we tried to develop a rapid subcutaneous immunotherapy (SCIT) model for *Humulus* allergy.

**Methods:**

BALB/c Mice were sensitized through the SC or IP route, with respective reference to previously established sensitization methods and allergen dosing, and challenged with nebulized *Humulus* pollen extract to induce asthma. To compare the two sensitization methods, airway hyperresponsiveness (AHR), inflammatory cell infiltration, allergen‐specific serum immunoglobulin (Ig)E (sIgE) levels, cytokine levels, and lung histopathology were assessed. The effects of SCIT (once every other day for 16 days) on airway inflammation, AHR, sIgE, and allergen‐specific serum IgG2a (sIgG2a) levels were evaluated by using the model established in this study.

**Results:**

Although mice sensitized by the SC or IP routes both showed AHR and airway inflammation, the SC route elicited significantly higher levels of sIgE, eosinophil inflammation, and T helper type 2 cytokines, compared with the IP route. SCIT in the treatment group significantly reduced the titers of sIgE, enhanced the titers of sIgG2a, and effectively alleviated pulmonary inflammation and AHR, compared with the vehicle group.

**Conclusions:**

The SC route can be used to establish a murine model of *Humulus* pollen allergy that recapitulates the characteristics of clinical allergic asthma. Short‐term SCIT can significantly improve symptoms and pathophysiology in asthmatic mice.

## Graphical abstract

The SC route can be used to establish a murine model of *Humulus* pollen allergy that recapitulates the characteristics of clinical allergic asthma. Short‐term subcutaneous immunotherapy can significantly improve symptoms and pathophysiology in asthmatic mice.

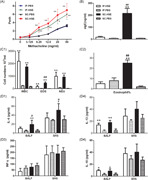

**Abbreviations**
AHR: airway hyperresponsiveness
*H. scandens*: *Humulus scandens*
HSE: *H. scandens* pollen extract
Ig: immunoglobulin
IP: intraperitoneal
Mch: methacholine
Penh: enhanced pause
SC: subcutaneous
SCIT: subcutaneous immunotherapy
SHS: splenocyte homogenate supernatants
sIgE: specific serum immunoglobulin E
sIgG2a: specific serum IgG2a

## 1 INTRODUCTION

Asthma is a chronic airway disease characterized by bronchial inflammation, airway hyperreactivity, and reversible airflow limitation; it is often triggered by inhalation of allergens.^1^ Pollens are important outdoor allergens associated with allergic asthma and seasonal rhinitis.^2^ *Humulus scandens* (*H. scandens*, also known as *Humulus japonicas* or Lücao in Chinese) is a widespread weed in East Asia.^3^ In Korea, *Humulus* pollen constitutes approximately 18% of total pollen during the pollination period.^4^ In China, Jia et al. first discovered that *Humulus* pollen is an important allergen in summer and autumn, second only to *Artemisia* pollen.^5^ Among individuals with fall hay fever, 59% were positive for *H. scandens*‐specific serum IgE.^6^ The symptoms of asthma triggered by *H. scandens* pollen are reportedly more serious than those induced by *Artemisia* pollen, although the mechanism underlying this effect has not been elucidated.^7^

Subcutaneous immunotherapy (SCIT) with crude *H. scandens* pollen extract (HSE) has been used in China for many years; this type of therapy can effectively reduce the symptom scores and medication scores of patients with rhinitis and asthma.^8^ However, the mechanisms underlying immunotherapy with HSE have remained unclear, thereby limiting the applications of this treatment method. Thus far, most patients with allergic asthma induced by *H. scandens* pollen are treated with inhaled and oral corticosteroids, despite the considerable side effects associated with long‐term use of corticosteroids.^9^ Therefore, it is necessary to establish an animal model of asthma with HSE and to study the mechanisms underlying successful SCIT.

Murine models of allergic asthma induced by *Humulus* pollen were previously established by Ya‐li et al.^10^ and Kong et al.^11^ through intraperitoneal (IP) injection and nasal drip stimulation. We originally planned to study the mechanisms underlying immunotherapy with HSE by using this model. However, A fair number of mice died after two nasal drops. We repeated the experiment more than three times with different anesthesia methods that used for nasal drip stimulation. Our results were similar, in that most mice died. The lung histopathology of surviving mice showed acute lung injury (see Supporting Information Material for representative experimental results). We speculated that these results may be attributed to direct stimulation of lung tissues with high concentrations of HSE during nasal drip challenge. Whether this is related to the fact that *H. scandens* is a kind of Chinese herbal medicine with antipyretic and detoxifying effects^12^ is unclear. Therefore, we attempted to use atomized inhalation for challenge, which is considered to be milder and has a lower fatality rate.^13^ In addition, IP injection is the most commonly used sensitization route, but some studies have suggested that sensitization by subcutaneous (SC) injection is more effective for eliciting high IgE levels and Th2 cytokines and for inducing allergic airway inflammation.^14^ For instance, Conejero et al.^15^ established a murine model of olive pollen allergic asthma through SC sensitization.

In the present study, we compared the IP and SC sensitization routes, with respective reference to previously established sensitization methods and allergen dosing,^10^, ^15^ to develop an optimized model of allergic asthma induced by HSE that can better simulate the immunological and pathological features of human allergic asthma.^16^ Using our asthma model, we then developed a model of rapid SCIT to simulate the process of clinical SCIT and explore the mechanisms of *Humulus* immunotherapy.

## 2 MATERIALS AND METHODS

### 2.1 Animals

Female, 6–8‐week‐old BALB/c mice (weight range, 18–20 g) were purchased from the Academy of Military Medical Sciences of China and maintained under a 12‐h light–dark cycle with free access to water and standard laboratory chow. All experimental animal procedures conformed to international standards of animal welfare and were approved by the Animal Experimentation Ethics Committee of Peking Union Medical College Hospital.

### 2.2 Allergen

*H. scandens* pollen was purchased from Beijing Key Laboratory of Precision Medicine for Diagnosis and Treatment on Allergic Diseases (Beijing, China). HSE was prepared using the extraction method previously described for *Artemisia vulgaris* pollen.^17^ Briefly, *H. scandens* pollen was defatted with acetone, extracted with 0.125 M ammonium bicarbonate (weight/volume = 1:20), dialyzed against distilled water, then aliquoted into vials (2.15 ml/vial) and freeze‐dried. Subsequently, freeze‐dried HSE was diluted in phosphate‐buffered saline (PBS) before use. The presence of endotoxin in the pollen and HSE was assessed using the limulus amebocyte lysate test, and the test results were qualified. The total protein concentration was determined by the Bradford method. The concentration of HSE dissolved in 200 μl PBS was 5.6 μg/μl (total protein, 1.12 mg/vial).

### 2.3 Experimental design

#### 2.3.1 Establishment of an optimized murine asthma model for *Humulus* pollen allergy

**Figure 1:**
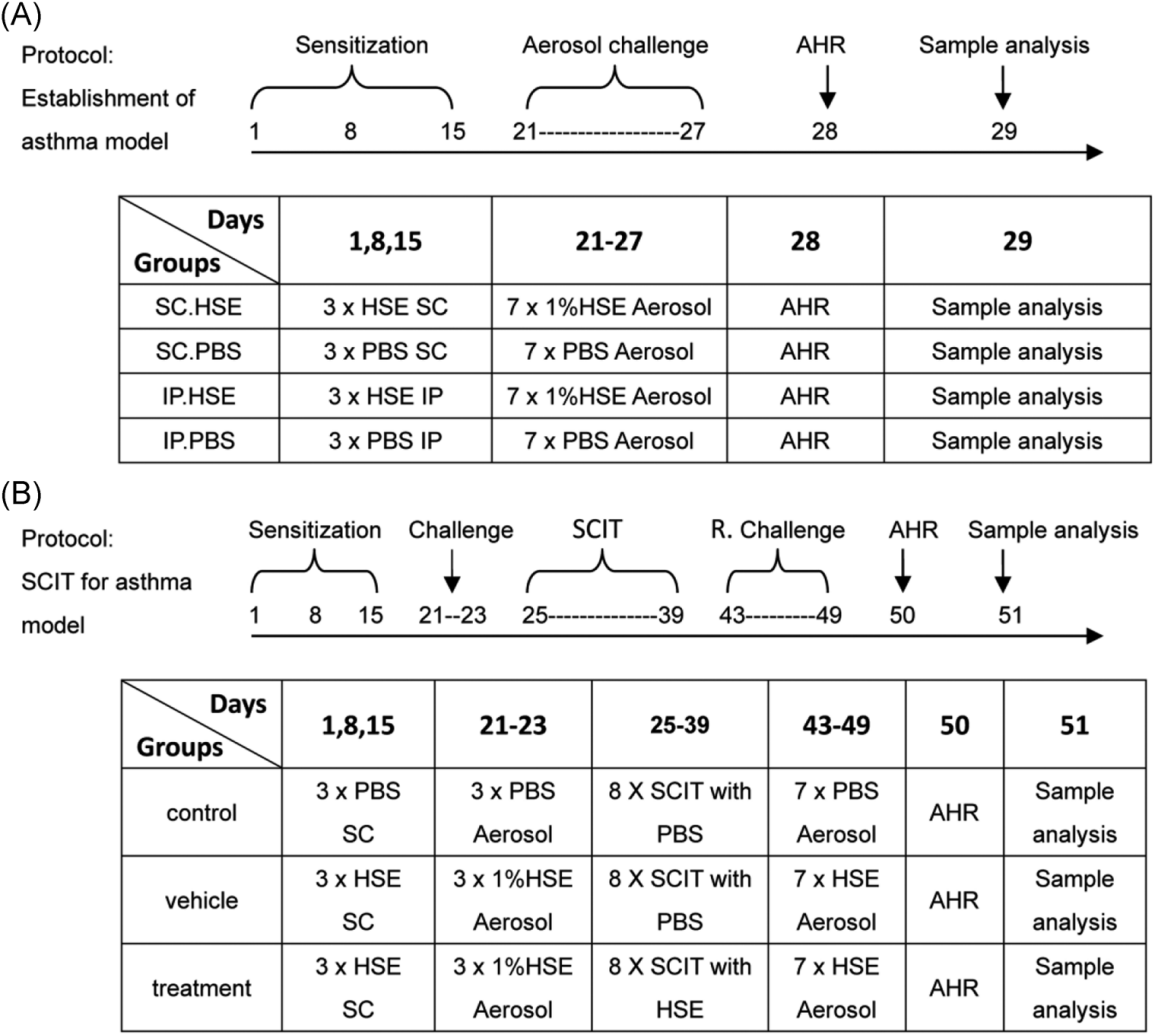
Overview of experimental design. Establishment of murine asthma model (A). Specific SCIT for murine asthma model (B). (A) Mice were sensitized by the SC or IP routes on days 1, 8, and 15 with HSE or PBS. Seven days later the last sensitization, all animals were challenged with aerosolized HSE (1% in saline) or saline (control group) for 30 min daily for 7 consecutive days. On day 28, AHR to Mch was assessed. On day 29, the mice were killed for further experiments. (B) Mice were sensitized on days 1, 8, and 15 by SC injection with 25 μg of HSE or PBS. Seven days after the last injection, all animals were placed in a plastic box and challenged with aerosolized HSE (1% in saline) or an equal volume of saline (control group) for 30 min daily for 3 consecutive days. On days 25–39, mice in the treatment group received eight SC injections of 300 μg of HSE every other day; the control and vehicle group animals received PBS. Mice were rechallenged with a 1% HSE aerosol on days 43–49 and on day 50, AHR to Mch was assessed. One day later, the mice were killed for further analysis. AHR, airway hyperresponsiveness, HSE, *H. scandens* pollen extract; IP, intraperitoneal; Mch, methacholine; PBS, phosphate‐buffered saline; SC, subcutaneous; SCIT, subcutaneous immunotherapy

Forty mice were randomly divided into four groups. Two different routes and doses were used for sensitization, based on studies by Ya‐li et al.^10^ and Conejero et al.^15^ (Figure 1A). Briefly, mice in the SC sensitization group (SC.HSE, *n* = 10) were sensitized via SC injection on the back of the neck with 25 μg of HSE adsorbed to alum adjuvant (Imject Alum; Pierce) in a volume of 200 μl. Mice in the SC control group (SC.PBS, *n* = 10) received SC injections of PBS adsorbed to alum adjuvant (200 μl). Mice in the IP sensitization group (IP.HSE, *n* = 10) were sensitized via IP injection—at approximately 1 cm from the intersection point of a line running between the thighs and the midline—with 300 μg of HSE adsorbed to alum adjuvant in a volume of 200 μl. Mice in the IP control group (IP.PBS, *n* = 10) received IP injections of PBS adsorbed to alum adjuvant (200 μl). Sensitization injections were administered at weekly intervals for 3 weeks. To induce asthma, beginning on day 21, sensitized and control mice were placed in a plastic box and challenged for 30 min daily via the airways with aerosolized 1% HSE or an equal volume of saline (control group) using a jet nebulizer (BARI Co., Ltd.), for 7 consecutive days. Airway hyperresponsiveness (AHR) to methacholine (Mch, Sigma‐Aldrich) was measured 24 h after the last challenge in all animals. On day 29, mice were anesthetized with 2% sodium pentobarbital; sera were then obtained for measurement of specific IgE levels and of IgE profiles binding to HSE. Additionally, bronchoalveolar lavage fluid (BALF) was prepared from the right lung for inflammatory cell enumeration and cytokine quantitation. Paraffin sections from the left lung were used for histological assessment. The spleen was homogenized for cytokine measurements.

#### 2.3.2 Rapid SCIT for induction of HSE allergy in murine asthma model

The SCIT model for *Humulus* pollen allergy was developed on the basis of the asthma model established as shown in Figure 1A, and in combination with methods described by Leonie et al.^18^ The detailed process was as follows (Figure 1B): 30 BALB/c mice aged 6–8 weeks were randomly divided into control, vehicle, and treatment groups (10 mice per group). Mice in the vehicle and treatment groups were sensitized by SC injection with 25 µg of HSE adsorbed to alum adjuvant on days 1, 8, and 15; they were then challenged with atomized HSE on days 21–23 to induce symptoms. SCIT was started on day 25, comprising eight treatments every other day. In the treatment group, mice received an SC injection of 300 µg of HSE (2 μg/μl, 150 μl) into the neck. Mice in the vehicle group received an SC injection of 150 μl of PBS. To investigate the effectiveness of SCIT, mice in the vehicle and treatment groups were rechallenged with an HSE aerosol on days 43–49. In parallel, control mice received saline, rather than HSE, at all steps. On day 50, AHR to Mch was assessed in all animals. One day later, all mice were killed. Sera were obtained for measurement of specific IgE levels and specific IgG2a levels; other samples for analysis were harvested as described above.

### 2.4 Assessment of AHR to Mch

AHR was assessed using a whole‐body plethysmography system (Enterprise EMKA Technologies), as described elsewhere.^19^ Unrestrained, spontaneously breathing mice were placed into chambers. The enhanced pause (Penh), a function of the maximal expiratory and inspiratory box pressure signals and the timing of expiration that is closely related to pulmonary resistance, was calculated. Mice were exposed for 2 min to nebulized PBS, then to increasing concentrations (3.125, 6.25, 12.5, 25, and 50 mg/ml) of nebulized Mch in PBS. After each nebulization, 3 min recordings were taken. Penh measurements were averaged for each Mch concentration.

### 2.5 Preparation of BALF and cell enumeration

After mice had been anesthetized, the trachea was surgically exposed and cannulated. The left lung was processed for paraffin section analysis. The right lung was lavaged three times with a single volume of warm PBS. The BALF was centrifuged at 400*g* for 10 min at 4°C; the supernatant was then stored at −80°C until cytokines were measured. Cell pellets from BALF were resuspended in PBS; leukocytes were classified and enumerated using an automatic blood analyzer (ADVIA2120, Siemens). Results were expressed as the number of each cell type per 1 ml of BALF.

### 2.6 Preparation of splenocyte homogenate supernatants (SHS)

Spleens were removed and promptly homogenized in 5 ml of ice‐cold radioimmunoprecipitation assay buffer with a mortar. Homogenates were centrifuged at 12,000*g* for 20 min at 4°C; supernatants were stored at −80°C until cytokines were measured.

### 2.7 Measurement of cytokine levels in BALF and SHS

The concentrations of interleukin (IL)‐4, IL‐13, interferon (IFN)‐γ, and IL‐10 in BALF and SHS were measured by sandwich enzyme‐linked immunosorbent assay (ELISA), in accordance with the manufacturer's instructions (eBioscience Co.). Values were interpolated from standard curves using recombinant cytokines and expressed in pg/ml.

### 2.8 Allergen‐specific serum IgE measurement

Allergen‐specific serum IgE levels were measured by ELISA. Briefly, wells of microtiter plates (Costar, Corning Inc.) were coated with 100 μl/well of HSE (500 μg/ml) and reference wells were coated with purified anti‐mouse IgE capture antibody (BD Biosciences Pharmingen). The next day, the plates were blocked and washed, and then 50 μl of each serum sample (diluted 1:5 in blocking buffer) was applied to sample wells. A series of eight two‐fold dilutions of purified mouse IgE (BD Biosciences Pharmingen) were used in conjunction with reference wells as standards; plates were then incubated overnight. Subsequently, they were treated with 100 μl of biotinylated monoclonal anti‐mouse IgE antibody (1 μg/ml, BD Biosciences Pharmingen) and horseradish peroxidase‐streptavidin (diluted 1:1000; BD Biosciences Pharmingen) for 1 h each at 37°C. Finally, 100 μl/well of TM Blue (Cwbiotech) was applied as a substrate, and the reactions were allowed to develop at room temperature for 20 min. The plates were read at 450 nm using an ELISA plate reader (BioTek, ELX800). A standard curve of murine IgE was used as a reference.

### 2.9 Allergen‐specific serum IgG2a measurements

Allergen‐specific serum IgG2a levels were measured by ELISA. Briefly, plates were coated with 100 μl of HSE (5 μg/ml) overnight at 4°C and reference wells were coated with antimouse IgG2a (0.5 μg/ml, BD Biosciences Pharmingen). After the wells had been blocked and washed, serum (diluted 1:1000) was added to HSE‐coated wells and purified IgG2a (BD Biosciences Pharmingen) was added to reference wells; the plates were incubated overnight at 4°C. Plates were subsequently washed and incubated with alkaline phosphatase‐labelled goat anti‐mouse IgG2a (diluted 1:2000; Southern Biotechnology Associates) for 2 h at room temperature. After plates had been washed, a solution of 4‐nitrophenyl phosphate (Roche) was added to each well. Absorbance at 450 nm was measured 1 h after the addition of substrate. A standard curve of murine IgG2a was used as a reference.

### 2.10 Lung histopathology

Mouse lungs were fixed with 10% formalin, and paraffin sections were prepared by the Histology Core at Peking Union Medical College Hospital. Hematoxylin and eosin staining was performed to examine inflammatory infiltrates; Alcian blue‐periodic acid‐Schiff staining (Sigma‐Aldrich) was performed to evaluate mucus production. To determine the severity of inflammatory cell infiltration, peribronchial and perivascular inflammatory cell infiltration was blindly evaluated using a five‐point scoring system, as previously described^17^: 0, no cells; 1, a few cells; 2, a ring of cells with 1‐cell depth; 3, a ring of cells with 2–4‐cell depth; 4, a ring of cells with >4‐cell depth. To evaluate the severity of mucus production, goblet cell hyperplasia in airway epithelium was evaluated semiquantitatively under a light microscope (20× magnification) in a blinded manner, using a five‐point grading system described previously.^17^ Alcian blue‐stained goblet cells were counted and expressed as the percentage of the total number of epithelial cells: 1, <25%; 2, 25%–50%; 3, 50%–75%; 4, >75%. The scoring of inflammatory cells and goblet cells was performed in at least three different fields for each lung section. Average scores were obtained from 10 mice.

### 2.11 Serum IgE antibody binding to HSE

IgE antibody binding to HSE in mouse sera was analyzed by sodium dodecyl sulfate poly‐acrylamide gel electrophoresis (SDS‐PAGE) and Western blot analysis. Briefly, 20 μg of HSE was separated by SDS‐PAGE with a NuPAGE® Novex 4%–12% Bis‐Tris precast gel (Invitrogen) under reducing conditions. After electrophoresis, proteins were transferred to a polyvinylidene difluoride membrane. The membrane was blocked with PBS containing 5% skim milk and 0.05% Tween‐20 for 2 h at room temperature, then separated into strips and incubated with the sera of sensitized or control mice (diluted 1:6) overnight at 4°C. Subsequently, polyvinylidene difluoride strips were washed with PBS containing 0.05% Tween‐20 and incubated for 2 h at room temperature with rat–anti‐mouse IgE (diluted 1:1000; Abcam). IgE‐binding proteins were detected using BM Chemiluminescence Blotting Substrate (Roche), in accordance with the manufacturer's instructions.

### 2.12 Statistical analysis

Data are presented as mean ± *SE* of the mean (SEM). All statistical analyses were performed using SPSS Statistics, version 19.0 (IBM Corp). Analysis of variance and Student's *t*‐test were used to assess differences between groups. Differences with *p* < .05 were regarded as statistically significant. All experiments were performed independently three times with similar results. Representative results from one of the three experiments are shown.

### 2.13 ETHICAL APPROVAL

All experimental animal procedures conformed to international standards of animal welfare and were approved by the Animal Experimentation Ethics Committee of Peking Union Medical College Hospital.

## 3 RESULTS

### 3.1 Effect of SC or IP route sensitization on airway inflammation and immunological response

**Figure 2:**
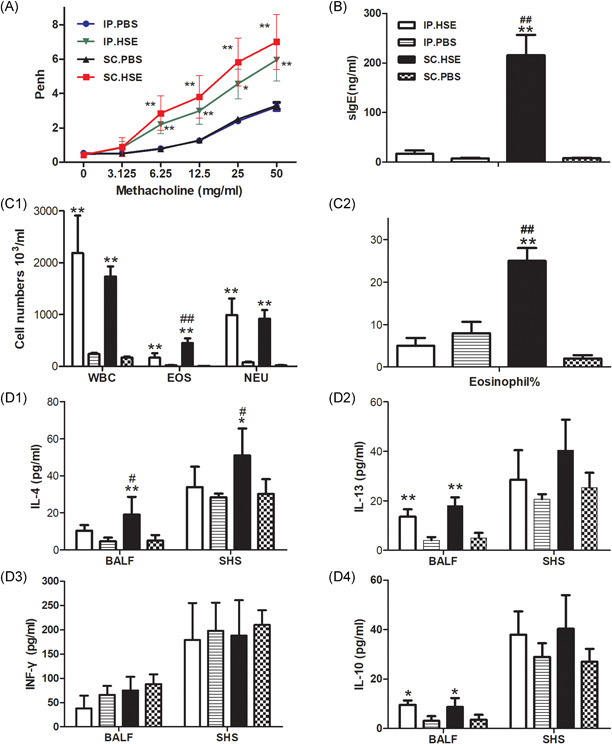
The effect of SC route or IP route sensitization on AHR, airway inflammation and immunological response. Mice were sensitized by SC route or IP route and aerosol challenged as described in Figure 1A. AHR to Mch (A): Penh dose–response curves to Mch were determined 24 h after the last challenge (on day 28). The mice were killed 1 day later for analysis of HSE‐specific IgE (B) in serum, inflammatory cell recruitment (C1) and percentages of eosinophils (C2) in BALF, Cytokine production of IL‐4 (D1), IL‐13 (D2), IFN‐γ (D3), and IL‐10 (D4) in BALF and SHS. Data are expressed as mean ± SEM of *n* = 10 mice per group. **p < .01, SC.HSE vs SC.PBS group, or IP.HSE vs IP.PBS group; *p < .05, SC.HSE vs SC.PBS group, or IP.HSE vs IP.PBS group; ^##^ *p* < .01, SC.HSE vs IP.HSE group; ^#^ *p* < .05, SC.HSE vs IP.HSE group. AHR, airway hyperresponsiveness, HSE, *H. scandens* pollen extract; IP, intraperitoneal; Mch, methacholine; PBS, phosphate‐buffered saline; SC, subcutaneous; SCIT, subcutaneous immunotherapy

**Figure 3:**
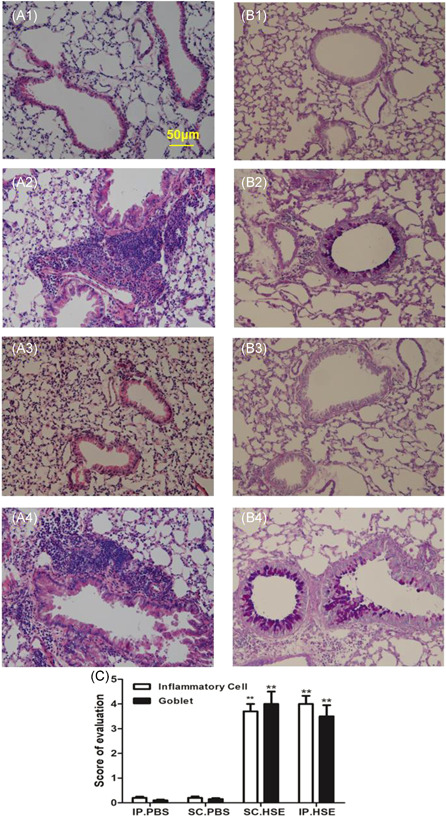
Inflammatory cell infiltration (A), mucus production (B) in lung tissue and scoring of inflammatory cells and goblet cells (C). Lung tissues were harvested 24 h after the last HSE challenge. H&E staining was performed to examine cell infiltration and Alcian blue‐periodic acid‐Schiff staining was performed to evaluate mucus production. Representative images for inflammatory cell infiltration (A1–A4) and goblet hyperplasia are shown (B1–B4): A1 and B1, IP.PBS group; A2 and B2, IP.HSE group; A3 and B3, SC.PBS group; A4 and B4, SC.HSE group. The scoring of inflammatory cells and goblet cells (C) was performed in at least three different fields for each lung section, and the data are expressed as mean ± SEM of *n* = 10 mice per group.**p < .01, SC.HSE vs SC.PBS group, or IP.HSE vs IP.PBS group. AHR, airway hyperresponsiveness, HSE, *H. scandens* pollen extract; IP, intraperitoneal; Mch, methacholine; PBS, phosphate‐buffered saline; SC, subcutaneous; SCIT, subcutaneous immunotherapy

Compared with the respective control groups, mice sensitized with HSE by either the IP or SC routes both developed severe AHR to Mch, characterized by significantly greater Penh values at 6.25, 12.5, 25 and 50 mg/ml of Mch inhalation (Figure 2A, *p* < .01 or *p* < .05). These changes were accompanied by recruitment of total white blood cells and inflammatory cell subpopulations (e.g., eosinophils and neutrophils) to the airways (Figure 2C1, *p* < .01, respectively); elevated levels of IL‐13 (Figure 2D2, *p* < .01) and IL‐10 (Figure 2D4, *p* < .05) in the BALF; inflammatory infiltrates around bronchi; and elevated mucus production (Figure 3C, *p* < .01). In contrast, IL‐13 (Figure 2D2) levels and IL‐10 (Figure 2D4) levels in SHS, and IFN‐γ (Figure 2D3) levels in both BALF and SHS, did not show any clear changes in HSE‐sensitized mice, compared with the respective control groups.

In addition, compared with mice in the SC.PBS group, mice sensitized with HSE by the SC route developed higher HSE‐specific serum IgE levels (Figure 2B, *p* < .01); higher numbers (Figure 2C1, *p* < .01) and percentages (Figure 2C2, *p* < .01) of BALF eosinophils; and higher production of IL‐4 in BALF (Fig. 2D1, *p* < .01) and SHS (Figure 2D1, *p* < .05). However, these characteristics were not observed in the IP.HSE group, compared with the IP.PBS group (Figure 2B, C1, C2, and D1).

Notably, HSE‐specific serum IgE levels (Figure 2B, *p* < .01); numbers (Figure 2C1, *p* < .01) and percentages (Figure 2C2, *p* < .01) of BALF eosinophils; and production of IL‐4 in BALF (Figure 2D1, *p* < .05) and SHS (Figure 2D1, *p* < .05) were all significantly higher in the SC.HSE group than in the IP.HSE group.

**Figure 4:**
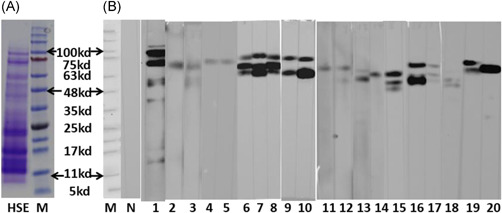
SDS‐PAGE (A) and Western blot analysis (B). (A) Representative image of SDS‐PAGE. Lane HSE: 20 μg protein profiles of *H. scandens* pollen extract. Lane M, molecular mass markers. (B) Western blot analysis analysis of the antigen‐binding characteristics of serum specific IgE antibodies with *H. scandens* pollen extract. Lane M, molecular mass markers; lane N, pooled sera from both control groups; lanes 1–10, sera from SC.HSE group animals; lanes 11–20, sera from IP.HSE group animals. HSE, H. scandens pollen extract; IP, intraperitoneal; SC, subcutaneous; SDS‐PAGE, sodium dodecyl sulfate poly‐acrylamide gel electrophoresis

Futhermore, the protein profile of HSE (determined by SDS‐PAGE) is shown in Figure 4A; the molecular weight of the proteins mainly ranged from 10 to 100 kDa. Most sera from animals in the SC.HSE and IP.HSE groups exhibited IgE reactivity against HSE proteins with apparent molecular masses of 48–100 kDa (Figure 4B). There were no significant differences in the distribution of immunoreactive bands between animals in the SC.HSE and IP. HSE groups. Pooled sera of animals in the control group showed no binding of IgE to HSE.

### 3.2 Effect of SCIT

**Figure 5:**
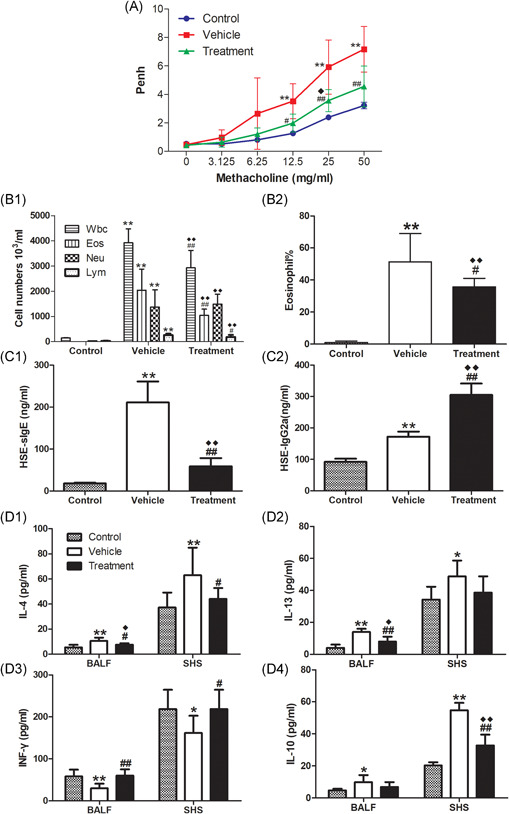
Effects of SCIT on HSE‐induced AHR, airway inflammation and immunological response. Mice were sensitized and challenged as described in Figure1 B. AHR to Mch(A): Penh dose–response curves to Mch were determined 24 h after the last challenge (on day 50). The mice were killed 1 day later for analysis of inflammatory cell recruitment (B1) and percentages of eosinophils (B2) in BALF, HSE‐specific IgE (C1) and IgG2a (C2) in serum and cytokine production of IL‐4 (D1), IL‐13 (D2), IFN‐γ (D3), and IL‐10 (D4) in BALF and SHS. Data are expressed as mean ± SEM of n = 10 mice per group. **p < .01, vehicle vs control group; *p < .05, vehicle vs control group; ^##^ *p* < .01, treatment vs vehicle group; ^#^ *p* < .05, treatment vs vehicle group. ^◆◆^ *p* < .01, treatment vs control group; ^◆^ *p* < .05, treatment vs control group. AHR, airway hyperresponsiveness, HSE, *H. scandens* pollen extract

**Figure 6:**
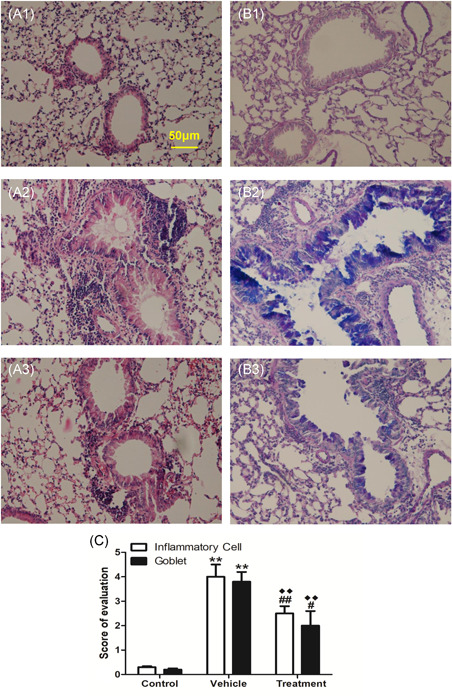
Effects of HSE SCIT on Inflammatory cell infiltration and goblet hyperplasia in the lungs of allergic mice. The experimental procedure is described in Figure 1B. H&E staining was performed to examine cell infiltration and Alcian blue‐periodic acid‐Schiff staining was performed to evaluate mucus production. Representative images for inflammatory cell infiltration (A) and goblet hyperplasia (B) are shown: A1 and B1, control group; A2 and B2, vehicle group; A3 and B3, treatment group. Inflammatory cell infiltration and goblet cell hyperplasia was blindly semiquantified (C). Scoring of inflammatory cells and goblet cells was performed in at least three different fields for each lung section, and the data were presented as mean ± SEM of *n* = 10 mice per group. **p < .01 vehicle vs control group; ^##^ *p* < .01, treatment vs vehicle group, ^#^ *p* < .05, treatment vs vehicle group. ^◆◆^ *p* < .01, treatment vs control group

Compared with the vehicle group, SCIT with HSE in the treatment group led to significant reduction of Penh values at 12.5, 25, and 50 mg/ml of Mch inhalation (Figure 5A, *p* < .05 or *p* < .01). It also reduced the infiltration of white blood cells (Figure 5B1, *p* < .01), eosinophils (Figure 5B1, *p* < .01), and lymphocytes (Figure 5B1, *p* < .05), as well as the percentage of eosinophils (Figure 5B2, *p* < .05) in BALF; inhibited HSE‐specific IgE production (Figure 5C1, *p* < .01) and enhanced HSE‐specific IgG2a production (Figure 5C2, *p* < .01); reduced IL‐4 levels in BALF (Figure 5D1, *p* < .05) and SHS (Figure 5D1, *p* < .05); enhanced IFN‐γ levels in BALF (Figure 5D3, *p* < .01) and SHS (Figure 5D3, *p* < .05), and reduced inflammatory infiltrates around the bronchi and diminished mucus production(Figure 6C, *p* < .01). Moreover, SCIT significantly reduced IL‐13 levels in BALF (Figure 5D2, *p* < .01) and IL‐10 levels in SHS (Figure 5D4, *p* < .01). Notably, SCIT did not significantly reduce allergen‐induced neutrophil accumulation in BALF (Figure 5B1).

Compared with the control group, the mice in the treatment group had larger Penh values. However, they only showed significantly greater Penh values at 25 mg/ml of Mch inhalation (Figure 5A, *p* < .05). The recruitment of total white blood cells and inflammatory cell subpopulations to the airways (Figure 5B1, *p* < .01, respectively); percentages of BALF eosinophils (Figure 5B2, *p* < .01); inflammatory infiltrates around bronchi, and mucus production (Figure 6C, *p* < .01) were all significantly higher in the treatment group than in the control group. Besides, compared with the control group, SCIT with HSE in the treatment group enhanced HSE‐specific IgE production (Figure 5C1, *p* < .01) and HSE‐specific IgG2a production (Figure 5C2, *p* < .01); enhanced IL‐4 levels (Figure 5D1, *p* < .05) and IL‐13 levels (Figure 5D2, *p* < .05) in BALF and IL‐10 levels in SHS (Figure 5D4, *p* < .01), but did not affect IFN‐γ levels in BALF and SHS (Figure 5D3).

## 4 DISCUSSION

*Humulus* pollen is an important allergen that causes asthma in East Asia. In this study, we showed that, compared with IP sensitization, SC sensitization provides a murine model that better recapitulates the typical clinical characteristics of allergic asthma. Using this model, we successfully developed a rapid SCIT model for HSE‐induced asthma. This SCIT model elucidated the mechanism of desensitization treatment, and provided a rationale for the use of HSE in clinical SCIT.

Compared with animals in the IP.HSE group, animals in the SC.HSE group showed significantly higher levels of allergen‐specific IgE and significantly higher quantity and proportion of eosinophils in BALF. These findings are consistent with the typical characteristics of allergic asthma patients.^16^ In our study, mice sensitized via the IP route showed an elevated number of eosinophils, but no change in their proportion. Following airway stimulation by atomized pollen, the number of inflammatory cells is globally elevated; thus, enhancement of absolute eosinophil number does not necessarily reflect the occurrence of a typical type I hypersensitivity reaction.

Allergic asthma is characterized by elevated expression of Th2 cytokines (i.e., IL‐4 and IL‐13) and reduced expression of Th1 cytokines (i.e., IFN‐γ).^20^ IL‐4 is a representative Th2 cytokine that can stimulate the proliferation of B and T cells; it enhances IgE synthesis^21^ and induces eosinophil infiltration into the airway.^22^ In this study, IL‐4 levels in the BALF and SHS were significantly higher in mice in the SC.HSE group, compared with mice in the SC.PBS and IP.HSE groups. This may explain why mice in the SC.HSE group showed higher HSE‐specific IgE levels and higher proportions of BALF eosinophils, while mice in the IP.HSE group did not. IL‐13 has been proposed to induce inflammatory cell infiltration into the airway, as well as mucus secretion and AHR.^23^ Compared with the respective control groups, BALF IL‐13 levels were significantly higher among mice in both SC.HSE and IP.HSE groups. Thus, IL‐13 may have promoted inflammatory cell infiltration, mucus secretion, and AHR in these animals, although IL‐4 levels were not elevated in the IP.HSE group. However, IFN‐γ levels in BALF and SHS of mice in the SC.HSE and IP.HSE groups only showed a slight downward trend, but no significant reduction.

The SC and IP sensitization routes may produce different immune responses because of the presence of different local antigen‐presenting cell populations at the site of antigen contact. In the skin, dendritic cells, which vary in quantity and phenotype, are reportedly found in atopic individuals in a manner that favors type 2 immune responses.^24^ In addition, mast cells, which can produce Th2 cytokines and amplify allergic response, are widely distributed in the dermis and act as sentinels at sites of initial antigen exposure.^25^ In contrast, in the peritoneal cavity, macrophages are found in large numbers and tend to favor the induction of Th1/Th0 immune responses.^26^

Our group previously found that the approximately 10 kDa component is a major allergen in *H. scandens* pollen among Chinese individuals; we also identified the complete amino acid sequence of this protein (GenBank: ADB97919.1). Except for this 10 kDa component, Park et al.^27^ reported that three protein components (13 kDa, 74 kDa, and 80 kDa) from *Humulus* pollen extract were bound to IgE, among sera from more than 50% of the patients. Sun et al.^28^ reported that 30.8% of patient serum samples exhibited binding interactions with 100.5‐kDa proteins in *Humulus* pollen. The immunoblotting experiments in the present study showed that the IgE antibodies of sensitized mice were mainly bound to relatively large fragments (48–100 kDa), and rarely bound to the 10‐kDa band. This finding is consistent with the study work of U.S. Eitzer et al.^29^ who found that mice sensitized with whole protein of timothy‐grass pollen did not produce IgE antibodies against PHLP1, the major allergen of timothy in humans. These findings suggested that mice and humans may be sensitive by somewhat different proteins.

Clinically, SCIT typically requires SC injection twice per week for 3–5 years. Considering differences in the life cycles of mice and humans, we used a rapid desensitization treatment method (eight treatments every other day) and effectively alleviated pulmonary inflammation and AHR in asthmatic mice. Our results were similar to those of a study in which mice received weekly treatments for a continuous 8‐week period.^30^ This short‐term and rapid desensitization method saves time and reduces the cost of mouse maintenance.

Despite the short treatment duration, allergen‐specific IgE was significantly lower in the treatment group than in the vehicle group; anti‐IgE therapy has been effective for some allergic diseases.^31^ In addition, SCIT has been shown to significantly enhance allergen‐specific IgG2a levels. Elevated levels of allergen‐specific IgG4 in humans (similar to IgG2a in mice) are accompanied by improvement of clinical symptoms.^32^ Therefore, the therapeutic effect of SCIT in our model may have been achieved by inhibiting production of allergen‐specific IgE and enhancing production of allergen‐specific IgG2a in serum, as in previous studies.^33^

SCIT corrected the imbalance of Th1/Th2 immune responses in asthmatic mice and converted the immune response to a Th1 type. This may be the underlying mechanism of desensitization treatment. IL‐10 is a pleiotropic cytokine that has both pro‐ and anti‐inflammatory effects during immune responses.^34^ In our study, IL‐10 levels were significantly increased in asthmatic mice and significantly decreased after SCIT, indicating that this cytokine may play a role in exerting pro‐inflammatory effects.

Notably, pulmonary inflammation and AHR to 25 mg/ml of Mch inhalation in mice of the treatment group were more serious than that of the control group, which suggests that although SCIT significantly improved the symptoms of asthmatic mice, it did not completely restore their condition to the level of healthy mice.

There were some limitations in this study. Changes in the systemic immune response were examined by analysis of SHS because of limited laboratory conditions; the sensitivity of this method is relatively low, we will try to coculture spleen cells with specific allergens in vitro to observe changes in the systemic immune response in the further studies.

In conclusion, we successfully developed a better murine asthma model for *Humulus* pollen allergy through SC sensitization and atomization challenge. Using this model, short‐term and rapid SCIT improved symptoms and pathophysiology in asthmatic mice. The murine models of asthma and SCIT for *Humulus* pollen allergy developed in this study may be valuable tools for studying the mechanisms of asthma and SCIT for airborne allergens, as well as for designing more efficacious therapies for allergic asthma.

## AUTHOR CONTRIBUTIONS

Guang P. Xi and Jia Yin designed the study. Guang P. Xi and Qian Zhang performed the experiments. Guang Peng Xi and Jia Yin wrote the paper. All authors edited the paper and provided substantial feedback.

## Data Availability

The data that support the findings of this study are available from the corresponding author upon reasonable request

## APPENDIX A1 FIGURE (A‐FIGURE) 1. EXPERIMENTAL PROCEDURE FOR IP SENSITIZATION AND INTRANASAL CHALLENGE

Thirty mice were randomly divided into vehicle group and PBS group (15 mice per group). Mice were sensitized on days 1, 8, and 15 by IP injection with 300 µg of HSE or PBS. On days 22 and 23, mice were challenged by intranasal administration of 30 µL (10 mg/ml) of HSE or saline (PBS group). On day 24, AHR to Mch was assessed. On day 29, the mice were killed for histopathological analysis.

## APPENDIX A2 **A‐FIGURE** 2**. MORTALITY IN MICE CHALLENGED BY NASAL HSE DRIP**

Mice were challenged twice by intranasal administration of HSE (Vehicle group) or saline (PBS group). The number of mice died after each nasal drop was recorded.

## APPENDIX A3 **A‐FIGURE** 3**. AHR TO MCH**

Twenty‐four h after the second intranasal challenge with HSE, Penh in response to inhalation of different doses of Mch was determined in surviving mice.

## APPENDIX A4 **A‐FIGURE** 4**. INFLAMMATORY CELL INFILTRATION OF LUNG TISSUE**

Lung tissues were harvested on day 25 and H&E staining was performed to examine inflammatory cell infiltration. Representative images of inflammatory cell infiltration are shown. A, PBS group; B, Vehicle group.
